# The impact of stress and earthquake-related trauma on fibromyalgia: a longitudinal study

**DOI:** 10.1007/s11845-025-03955-y

**Published:** 2025-04-05

**Authors:** Muhammed Aslan, Irada Ibramkhalilova, Melis Mutlu, Fatih Albayrak, Pinar Gunel, Bunyamin Kisacik

**Affiliations:** 1 Department of Internal Medicine, Sanko University, Gaziantep, Turkey; 2 Department of Rheumatology, Sanko University, Gaziantep, Turkey; 3 Department of Rheumatology, Gaziantep University, Gaziantep, Turkey; 4 Department of Biostatistics, Sanko University, Gaziantep, Turkey

**Keywords:** Earthquake, Fibromyalgia, Natural disaster, Quality of life, Stress, Trauma

## Abstract

**Background:**

Fibromyalgia (FM) is a chronic disorder characterized by widespread pain, fatigue, and psychological symptoms. While psychological trauma is known to affect FM severity, the impact of natural disasters, such as earthquakes, remains insufficiently studied.

**Aim:**

This study aimed to evaluate the effects of earthquake-related trauma on fibromyalgia severity and quality of life.

**Methods:**

A total of 100 patients diagnosed with fibromyalgia per the American College of Rheumatology 2016 criteria were enrolled. All participants experienced the February 2023 earthquakes in Turkey. Disease activity and quality of life were assessed using the Fibromyalgia Impact Questionnaire (FIQ) at 1 and 6 months post-earthquake. The Impact of Event Scale-Revised (IES-R) was employed to measure psychological trauma levels. Statistical analyses included paired *t*-tests and Pearson’s correlation coefficients.

**Results:**

FIQ scores significantly improved from 1 month (74.97 ± 20.15) to 6 months (67.25 ± 16.39) post-earthquake (*p* < 0.001). A strong positive correlation was found between IES-R and FIQ scores at 1 month (*r* = 0.636, *p* < 0.01) and 6 months (*r* = 0.411, *p* < 0.01). Subgroups with home or family loss showed more pronounced FIQ scores, while groups exposed to new stress factors did not show significant changes over time (*p* = 0.307).

**Conclusions:**

This study demonstrates that earthquake-related trauma exacerbates fibromyalgia symptoms, highlighting the necessity of integrating psychological and stress management interventions in the treatment of FM, particularly in disaster-affected regions.

## Introduction

Fibromyalgia is characterized by widespread musculoskeletal chronic pain, poor sleep, fatigue, frequent psychological difficulties, and multiple tender points on physical examination [1, 2]. The etiology of fibromyalgia is unknown but may involve neuropsychiatric vulnerability [3]. There are many factors that contribute to fibromyalgia. Some of these are emotional factors, psychological factors, sudden shock, depression, and traumatic experiences. There is evidence of the relationship between traumatic experiences and prevalence of fibromyalgia diagnosis [4, 5]. Trauma is an event or situation that threatens a person's physical and/or spiritual integrity. Earthquake trauma is the trauma experienced by people who are directly or indirectly exposed to an earthquake. An earthquake is a natural disaster and can evoke emotions such as shock, surprise, anger, helplessness, powerlessness, loss of confidence, loss of control, and fear of death.

Turkey is located on the highly seismically active Anatolian Plate, on which massive earthquakes occurred throughout history. Since the year 1900, 20 earthquakes with a magnitude of over 7 have occurred in these lands, placing Turkey among the top-listed countries affected by earthquakes. On February 6, 2023, two independent earthquakes occurred at Gaziantep Şehitkamil Sofalaca epicenter at 04:17 local time with Mw = 7.7 magnitude and at Kahramanmaraş Ekinözü epicenter at 13:24 local time with Mw = 7.6 magnitude. On February 20 th, 2023, another earthquake with a magnitude of Mw 6.4 occurred, with the epicenter of Yayladağı, Hatay, at 20:04 local time. These earthquakes, each of which was unprecedented in recent history in terms of magnitude and coverage, caused major devastation in a total of 11 provinces. According to official statements, approximately 50,000 people lost their lives in these earthquakes. Like all people living in the region, fibromyalgia patients were exposed to traumatic stress due to the earthquake. The aim of this study is to investigate the effect of traumatic stress on fibromyalgia patients.

## Materials and method

This study was carried out in the rheumatology department clinic of a tertiary hospital. The study was conducted on a group of patients who were diagnosed with fibromyalgia according to the American College of Rheumatology (ACR) 2016 Diagnostic Criteria and were exposed to the earthquakes in our country on February 6, 2023. The patients’ demographic information, their physical effects from the earthquake, and whether they were exposed to a new stress factor after the earthquake were questioned and recorded using a study-specific questionnaire. The study was carried out in accordance with the ethical principles and the Helsinki Declaration. Informed consents of the participants were obtained. The study protocol was approved by the Local Ethics Committee (Sanko University Hospital, Approval Date: 04 October 2023, Number: 2023/08–9).

The patients were evaluated with the Fibromyalgia Impact Questionnaire (FIQ) as part of our routine examination at their visit approximately 1 month after the earthquake, and when these patients came to their routine 6 th month visit, the Fibromyalgia Impact Questionnaire (FIQ) was applied again to determine the disease activity and the effect of fibromyalgia on quality of life. In addition, they were asked whether they had been exposed to a new stress factor after the earthquake at the 6 th month visit.

Patients were given the Fibromyalgia Impact Questionnaire (FIQ), a 10-question questionnaire designed by Burckhardt et al. measuring patient physical function, work status, depression, anxiety, sleep, pain, stiffness, fatigue, and well-being [6]. Based on the responses to this survey, patients were evaluated with a score between 0 and 100. A fibromyalgia patient’s score must be at least 50, and an increase in this score is considered to indicate increased physical disability and decreased quality of life.

The Revised Event Impact Scale was used to measure how difficult an individual's life was after a traumatic stress experience. The Revised Event Impact Scale is a 22-item measurement tool designed by Weiss DS and Marmar CR in 1997 to measure the level of psychological trauma and post-traumatic stress [7]. The higher the total score received by the patients from the 22 items they scored, the more they were considered to be affected by the event.

Quantitative data obtained from the research were defined with mean and standard deviation; qualitative data were defined with number and percentage values. The conformity of quantitative data to normal distribution was evaluated with the Shapiro Wilk test. A dependent groups *t*-test was used in the first and sixth month comparisons. Pearson’s correlation coefficient was used to evaluate the relationship between scale scores. In all evaluations, *p* < 0.05 was accepted as statistically significant.

## Results

A total of 100 fibromyalgia patients were included in the study. Demographic information of the patients is shown in Table 1. The mean age of the patients was 42.22 ± 10.75 years. These findings provide a comprehensive demographic and clinical profile of the study population, highlighting the predominance of women, a high prevalence of housewives, and significant exposure to earthquake-related traumatic events (Table 2).

**Table 1:** Demographic characteristics of the patients

Variable	*n* (%)
Gender	
Male	10 (10)
Female	90 (90)
Marital status	
Married	90 (90)
Single	10 (10)
Education level	
Primary	33 (33)
Middle	11 (11)
High school	35 (35)
University or higher	15 (15)
Others	6 (6)
Occupational status	
Housewife	74 (74)
Self-employed	14 (14)
Worker	5 (5)
Civil Servant	4 (4)
Other	2 (2)
Health	
Chronic illness	26 (26)
Psychiatric disorder	21 (21)
Psychiatric medication use	31 (31)
Earthquake exposure	
Lost family	16 (16)
Lost home	16 (16)
Injured	2 (2)

**Table 2:** Comparison FIQ scores 1 st month after earthquake and 6 th month after earthquake

	1 st month after earthquake (Mean ± SD)	6 th month after earthquake (Mean ± SD)	*p* value
FIQ score	74.97 ± 20.15	67.25 ± 16.39	< 0.001*

*SD* standard deviation

^*^t-test

Pearson’s correlation analysis revealed significant relationships among IES-R (Impact of Event Scale-Revised) scores, FIQ scores at 1 month, and FIQ scores at 6 months (Table 3).

**Table 3:** Correlation between IES-R scores and FIQ scores

	IES-R scores	FIQ scores at 1 month	FIQ scores at 6 months
IES-R scores	-		
*r*		0.636	0.411
*p*		< 0.001	< 0.001
FIQ scores at 1 month			
r			0.520
*p*			< 0.001

These findings indicate a significant decrease in the impact of fibromyalgia over time, as measured by the FIQ scores. Additionally, the positive correlations suggest that higher stress or trauma levels (as measured by IES-R) are associated with greater fibromyalgia severity at both time points (Table 4).

**Table 4:** Comparison of 1 st and 6 th month FIQ scores of fibromyalgia patients after earthquake according to examined subgroups

Group	FIQ score 1 st month after earthquake (mean ± SD)	FIQ score 6 th month after earthquake (mean ± SD)	*p* value
With new stress factor	64.43 ± 22.75	67.66 ± 14.62	0.307
Without new stress factor	77.77 ± 18.57	67.14 ± 16.91	< 0.001
With psychiatric diagnosis	82.78 ± 13.59	71.52 ± 13.24	0.001
Without psychiatric diagnosis	72.89 ± 21.15	66.11 ± 17.02	0.002
With family loss	81.88 ± 20.18	72.25 ± 18.29	0.025
Without family loss	73.65 ± 20.00	66.30 ± 15.94	0.001
Home loss	83.35 ± 14.55	70.71 ± 16.58	0.007
No home loss	73.37 ± 20.74	66.59 ± 16.37	0.001

## Discussion

There are many factors that affect the disease in fibromyalgia patients, such as stress and anxiety, sleep disorders, physical and emotional traumas, inactivity or excessive exercise, hormonal changes, diet, and weather conditions. The number of studies on the effects of severe natural events on disease is low. In this study, we found that earthquake exacerbate the disease in fibromyalgia patients, but this effect decreases over time.

The pathophysiology of fibromyalgia involves a number of factors, including abnormalities in the neuroendocrine and autonomic nervous systems, genetic factors, psychosocial variables, and stress factors. Fibromyalgia is often affected by conditions such as anxiety, depression or psychological trauma. Conditions such as low serotonin levels as well as disturbances in cytokine levels are speculated to be the underlying cause. Russell et al. found that relative to healthy controls, fibromyalgia patients are characterized by low blood serum levels of serotonin and low cerebrospinal fluid levels of metabolites of serotonin, norepinephrine, and dopamine [8]. Luke O'Mahony et al. showed that there are significant differences in the peripheral blood cytokine profiles of fibromyalgia patients compared to the healthy group and touched upon the potential role of neuroinflammation in the pathogenesis of fibromyalgia [9].

There is a study by Salaffi et al. on the health-related quality of life of fibromyalgia patients following the massive earthquakes that occurred in central Italy in August and October 2016 [10]. This study, based in Italy, compared 55 fibromyalgia patients exposed to an earthquake with a control group of 49 fibromyalgia patients who were not exposed to an earthquake, and concluded that natural disasters such as earthquakes can significantly affect key areas such as pain, fatigue, sleep, and overall quality of life in fibromyalgia patients. Usui et al. showed that traumatic stress caused by the earthquake in Japan on March 11, 2011, aggravated fractures in fibromyalgia patients [11]. Similar to these studies, we also found that the disease worsened after trauma exposure. Although disease activity was quite high in the first month after the earthquake, we found that disease activity decreased in patients in the 6 th month visits as the effect of trauma decreased.

In addition to earthquakes, similar results have been observed in post-traumatic quality of life in other natural disasters such as tsunamis and hurricanes. A study conducted by Johan Siqveland et al. on a total of 58 Norwegian tsunami victims in Khao Lak, Thailand, during the 2004 Southeast Asian Tsunami showed that post-traumatic stress and depression were negatively associated with quality of life after a natural disaster [12].

This study was conducted with a sufficient number of patients, but it has some limitations: Firstly, we do not know the FIQ scores of the patients included in the study before the earthquake. Knowing these could have shown the effects of the earthquake better. Secondly approximately 1.5 years have passed since the earthquake and we do not have the long-term results of the patients. Thirdly we did not include healthy individuals as a control group in the study. Including healthy individuals in the study could have better demonstrated the effects of trauma on fibromyalgia patients.

As a result, many natural disasters can affect fibromyalgia. Among these, the earthquake, which has a more important place in terms of scope and destructiveness, causes a significant aggravation in fibromyalgia. It is useful to take these factors into consideration in the follow-up and treatment of these patients.

## Data Availability

The data supporting the findings of this study are available in [repository name] at [DOI or URL]. Alternatively, the data can be obtained from the corresponding author upon reasonable request.
